# Targeting Kaposi’s sarcoma associated herpesvirus encoded protease (ORF17) by a lysophosphatidic acid molecule for treating KSHV associated diseases

**DOI:** 10.3389/fcell.2023.1060156

**Published:** 2023-01-17

**Authors:** Misbahuddin M Rafeeq, Alaa Hamed Habib, Alaa F. Nahhas, Najat Binothman, Majidah Aljadani, Jawaher Almulhim, Ziaullah M Sain, Mohammad Zubair Alam, Norah A Alturki, Qamre Alam, Manish Manish, Rajnish Kumar Singh

**Affiliations:** ^1^ Department of Pharmacology, Faculty of Medicine, Rabigh, King Abdulaziz University, Jeddah, KSA; ^2^ Department of Physiology, Faculty of Medicine, King Abdulaziz University, Jeddah, KSA; ^3^ Biochemistry Department, Faculty of Science, King Abdulaziz University, Jeddah, KSA; ^4^ Department of Chemistry, College of Sciences and Arts, King Abdulaziz University, Rabigh, Saudi Arabia; ^5^ Department of Biological Sciences, King Faisal University, Alahsa, KSA; ^6^ Department of Microbiology, Faculty of Medicine, Rabigh, King Abdulaziz University, Jeddah, KSA; ^7^ Pre-Clinical Research Unit, King Fahd Medical Research Center, King Abdulaziz University, Jeddah, Saudi Arabia; ^8^ Department of Medical Laboratory Sciences, Faculty of Applied Medical Sciences, King Abdulaziz University, Jeddah, Saudi Arabia; ^9^ Clinical Laboratory Science Department, College of Applied Medical Science, King Saud University, Riyadh, Saudi Arabia; ^10^ Medical Genomics Research Department, King Abdullah International Medical Research Center, King Saud Bin Abdulaziz University for Health Science, Riyadh, Saudi Arabia; ^11^ School of Computer and Integrative Sciences, Jawaharlal Nehru University, New Delhi, India; ^12^ Molecular Biology Unit, Institute of Medical Sciences, Banaras Hindu University, Varanasi, Uttar Pradesh, India

**Keywords:** KSHV, protease, ORF17, reactivation, malignancies

## Abstract

Kaposi’s sarcoma associated herpesvirus (KSHV) is causative agent of Kaposi’s sarcoma, Multicentric Castleman Disease and Pleural effusion lymphoma. KSHV-encoded ORF17 encodes a protease which cleaves -Ala-Ala-, -Ala-Ser- or -Ala-Thr-bonds. The protease plays an important role in assembly and maturation of new infective virions. In the present study, we investigated expression pattern of KSHV-encoded protease during physiologically allowed as well as chemically induced reactivation condition. The results showed a direct and proportionate relationship between ORF17 expression with reactivation time. We employed virtual screening on a large database of natural products to identify an inhibitor of ORF17 for its plausible targeting and restricting Kaposi’s sarcoma associated herpesvirus assembly/maturation. A library of 307,814 compounds of biological origin (A total 481,799 structures) has been used as a screen library. 1-oleoyl-2-hydroxy-sn-glycero-3-phospho-(1′-myo-inositol) was highly effective against ORF17 in *in-vitro* experiments. The screened compound was tested for the cytotoxic effect and potential for inhibiting Kaposi’s sarcoma associated herpesvirus production upon induced reactivation by hypoxia, TPA and butyric acid. Treatment of reactivated KSHV-positive cells with 1-oleoyl-2-hydroxy-sn-glycero-3-phospho-(1′-myo-inositol) resulted in significant reduction in the production of Kaposi’s sarcoma associated herpesvirus. The study identified a lysophosphatidic acid molecule for alternate strategy to inhibit KSHV-encoded protease and target Kaposi’s sarcoma associated herpesvirus associated malignancies.

## Introduction

Kaposi’s Sarcoma (KS) was first identified as a skin cancer that affected elderly Mediterranean men (Cesarman et al., 2019). This was followed by its identification as the most common neoplasm of AIDS infected people (Rihana et al., 2018; Cesarman et al., 2019). Presence of herpesvirus like particle were demonstrated by electron microscopy in Kaposi’s sarcoma samples in subsequent years (Goldsmith and Miller, 2009). Emergence of KS after renal transplant and involvement of cytokines in KS biology led the foundation to hypothesize role of host immune system and viral contribution in KS biology (Raeisi et al., 2013; Alomari and Totonchy, 2020). Kaposi’s sarcoma associated herpesvirus (KSHV) was discovered in 1994 by differential display PCR methods and in 1996, the complete sequence of KSHV genome was reported (Chang et al., 1994; Moore and Chang, 1995; Antman and Chang, 2000). KSHV belongs to γ-Herpesviridae family, and its genome consist of nearly 170 KB double stranded DNA with a terminal repeat sequence at both the ends (Munz, 2018). Later, KSHV infection was also reported in association with Multicentric Castle disease (MCD), Pleural effusion lymphoma (PEL) and Kaposi Sarcoma Inflammatory Cytokine syndrome (KICS) (Uldrick et al., 2012; Karass et al., 2017; Narkhede et al., 2018). Once KSHV infects a host cell, its genome undergoes several processing before it establishes latency (Purushothaman et al., 2016). In brief, KSHV genome undergoes extensive epigenetic rearrangement leading to the silencing of majority of KSHV genes (Purushothaman et al., 2016). Among the epigenetic changes, the most important modifications include methylation and acetylation of histone H3 proteins (Gunther and Grundhoff, 2010; Juillard et al., 2016; Campbell et al., 2020). The KSHV genome also undergoes circularization where both the terminal repeats join end by end to create a circular KSHV DNA (Uppal et al., 2015; Juillard et al., 2016). In the latently infected KSHV-positive cells, only few KSHV-encoded genes are expressed, which are essential for maintenance of latency (Parravicini et al., 2000; Jenner et al., 2001). The KSHV-encoded latency associated nuclear antigen (LANA, encoded by ORF73) is one of the major proteins which starts expressing after initial infection and maintain a good level of protein throughout latent phase of its life cycle (Ballestas and Kaye, 2011; Uppal et al., 2014). KSHV-encoded LANA, vCyclin and vFLIP are expressed from the same polycistronic operon, however, their expression at the transcript or protein remains different (Toth et al., 2010; Kumar Singh et al., 2021). This further indicate role of post-transcriptional and post-translational regulation for the expression of KSHV encoded genes (Campbell and Izumiya, 2012; Butnaru and Gaglia, 2018; Singh et al., 2018). The LANA protein is essential for attaching viral episomal DNA with host chromosome where its binds with its N-terminal region with KSHV episome and with its C-terminal region with host chromosome (Lu et al., 2012; Vazquez Ede et al., 2013). LANA is considered as the master regulator for the maintenance of latency and oncogenic transformation of the infected cells (Uppal et al., 2014; Singh et al., 2019). LANA is known for its potential to degrade bonafide tumor suppressor proteins such as P53 and retinoblastoma (Cai et al., 2006; Hume and Kalejta, 2009). The counterpart of KSHV-encoded LANA is replication and transcriptional activator (RTA) protein encoded by ORF50 of KSHV genome (Staudt and Dittmer, 2007). Several factors including chemical inducers of reactivation, radiation, hypoxia, or other stress conditions are known to activate expression of RTA (Aneja and Yuan, 2017). RTA in turns activate expression of KSHV-encoded genes required for replication of KSHV DNA as well as assembly and maturation of KSHV virions (Yan et al., 2019). KSHV reactivation represents an important event of KSHV life cycle which allows it to multiply and infect new cells and is highly associated with pathogenesis caused by KSHV (Ganem, 2010).

Treatment of KSHV associated diseases depends on several factors which include both immune competency of infected individuals as well as complexity of the underlying pathogenic condition (Sullivan et al., 2009; Coen et al., 2014). In many cases, a systemic administration of antiviral drugs is recommended while in other cases a localized delivery of antiviral drugs or surgical removal of infected tissue is performed (Moore and Chang, 2011; Lurain et al., 2018; Chen et al., 2019). Radiotherapy is also an alternate treatment approach for KSHV-associated pathogenic conditions (Bhutani et al., 2015). The major antiviral chemotherapeutic agents used for KSHV-associated pathogenic conditions are vincristine, vinblastine, bleomycin and alitretinoin, which are well established anti-tumor medications (Sissolak and Mayaud, 2005; Mlombe, 2008; Coen et al., 2014).

In the present study, we investigated effect of physiological allowed condition of hypoxia and chemical inducers of KSHV reactivation on the expression of KSHV-encoded protease (ORF17). Hypoxia, a physiological possible condition was able to up-regulate expression of KSHV-encoded protease. The up-regulation was enhanced in the presence of chemical inducers of KSHV reactivation, suggesting a synergistic effect of hypoxia and chemical inducers on KSHV reactivation. Interestingly, we observed that ectopic expression of hypoxia inducible factor 1 alpha (HIF1α) can also up-regulate expression of KSHV-encoded ORF17. We further screened a large library of natural compounds as an inhibitor against KSHV-encoded protease to target KSHV reactivation. The screening of natural compounds identified several compounds for possible interaction and inhibition of KSHV-encoded protease. Lysophosphatidic acid (Lyso 18:1/1-oleoyl-2-hydroxy-sn-glycero-3-phospho-(1′-myo-inositol)) showed highest docking score in the molecular simulation experiment. A subtoxic level of Lysophosphatidic acid (Lyso 18:1/1-oleoyl-2-hydroxy-sn-glycero-3-phospho-(1′-myo-inositol)) was used to investigate effect on production of KSHV and was able to inhibit KSHV production in reactivation in *in-vitro* conditions.

## Materials and methods

### Cell culture, hypoxic treatment, plasmid constructs and transfection

KSHV positive body cavity lymphoma cell lines BC3 was obtained from Prof. Erle S Robertson at University of Pennsylvania, Philadelphia, USA. and GFP-HIF1α plasmid and Supercos-GB22 (KSHV genomic region 85,000-102000bp cloned in Spercos bacmid) were also kind gift from Prof. Erle S Robertson at University of Pennsylvania. The cell lines were maintained in RPMI medium supplemented with 10% fetal bovine serum and appropriate antibiotics at 37°C/5% CO_2_ 2 in an humified CO_2_ incubator. Hypoxic treatment was performed by incubating cells in hypoxic chamber at the oxygen concentration of 1%. The electroporation of plasmid in cells were carried out using Bio-Rad Gene Pulser Xcell electroporator system. Briefly, 10 million cells were pelleted down followed by washing with phosphate buffer saline followed by pelleting down by centrifugation at 1,500 rpm for 5 min. The cell pellets were resuspended in 0.5 ml PBS and mixed with 10 µg of plasmid. Followed by transferring the whole content in a new electroporation cuvette. Electroporation was performed by providing electric shock at 350 V and 975 µF capacitance. Electroporation cells were transferred in a well of 6-well plate in a total medium volume of 2 ml and transferred to CO2 incubator overnight followed by transferring in a T25 flask next morning. For the lentiviral based transduction, the control or ShRNA encoding plasmids was co-transfected along with helper plasmids into the HEK293T cells. The lentivirus production was stimulated by adding media containing sodium butyrate. The supernatant was collected and the lentiviruses were concentrated by high-speed centrifugation. The lentiviral transduction was performed in the presence of 4 μg/ml polybrene in a 50 ml tube in the total volume of 1 ml. The cells were the pelleted down and cultured in medium containing 2 μg/ml puromycin for selection of stably transduced cells.

### RNA isolation, cDNA synthesis and real time PCR

RNA from cell lines were isolated using TRIzol reagent by standard chloroform extraction method. Briefly, cells were pelleted down by centrifugation at 1,500 rpm for 5 min. Cells were lysed directly by adding 1 ml of trizol reagent and pipetting several times. The lysed cells along with TRIzol reagent were incubated at room temperature for 10 min 200 µl of chloroform per 1 ml of Trizol used was added followed by vigorous shaking for 10 times. The mixture was centrifuged at 13,200 rpm for 10 min at 4°C. The separated top aqueous phase was transferred to a new tube followed by RNA precipitation by adding 0.5 ml isopropanol. The RNA pellets were washed with 75% ethanol and semidried at room temperature. RNA pellets were dissolved in RNase free water by pipetting up and down followed by incubating at 55°C for 5 min 2 µg of RNA was used to synthesize cDNA using RevertAid First strand synthesis kit according to manufacturer protocol. cDNA was diluted 10 times and 2 µl (10 ng/μl) was used per reaction in the real-time PCR experiment. GAPDH was used endogenous control. The sequence of primers used in this study are as follows a) PDK1 Forward primer: 5′-GGA​TCA​GAA​ACC​GAC​ACA​AT-3’; PDK1 Reverse primer 5′-ACA​TTC​TGG​CTG​GTG​ACA​GG-3’ (product size 100 bp) b) P4HA1 Forward primer 5′-GTT​GGG​CAT​CCA​GTA​AAT​GC-3’; P4HA1 Reverse primer 5′-AGG​ACC​AGA​TTC​TCC​AAC​TC-3’ (product size 77 bp) c) ORF17 Forward primer (set 1) 5′-CGG​ATT​ATC​TCC​CAG​TCA​CA-3’; ORF17 Reverse primer (set 1) 5′-CTT​GAA​ATA​GAC​CCA​GTG​TC-3’ (product size 90 bp) d) ORF17 Forward primer (set 2) 5′-GGG​CGC​CGA​CGC​GGC​ACA​GT-3’; ORF17 Reverse primer (set 2) 5′-GGC​GCG​CTC​CGA​CTT​AGA​TA-3’ (product size 90 bp) e) GAPDH Forward primer 5′-ACC​CAG​AAG​ACT​GTG​GAT​GG-3’; GAPDH Reverse primer 5′- TCA​GCT​CAG​GGA​TGA​CCT​TG-3’ (product size 124 bp).

### Western blot and immunofluorescence

Cells were lysed in radio-immunoprecipitation buffer (50 mM Tris-HCl, 150 mM NaCl, 1 mM EDTA, 1% (v/v) NP-40) for 1 h with vertexing at regular interval of 10 min. The lysed cells were centrifuged at 4°C for 20 min at 15,000 rpm to remove cell debris. The clear supernatant was transferred to a new micro centrifuge tube. Concentration of proteins were measured by standard Bradford reagent. Equal amounts of proteins were separated on 10% polyacrylamide gel followed by transferring to nitrocellulose membrane. The membrane was stained with ponceau reagent to confirm the transfer of proteins. The membrane was washed witht tris-buffered saline containing tween-20 followed by blocking with 5% skimmed milk for 1 h at room temperature. The membrane was incubated overnight with primary antibody against PDK1 (Santa Cruz Biotechnology, sc-515944) or GAPDH (Santa Cruz Biotechnology, sc-47724) followed by washing with TBST for times, 5 min each. IR -conjugated secondary antibodies were used to probe proteins on Odyssys scanner. Immunofluorescence experiments were performed as described earlier (Kumar Singh et al., 2021). In brief, KSHV-positive BC3 cells were reactivated by treatment with TPA and butyric acid. The reactivated cells were collected by centrifugation, washed and semi-dried in a well of 8-well slides. The cells were fixed by dipping in 4% paraformaldehyde for 15 min at room temperature followed by washing with PBS. The permealization and blocking were performed in a single step by adding 3% bovine serum albumin supplemented with 0.1% triton X-100. The permealization and blocking was performed for 1 h at room temperature. Incubation with primary antibody was performed overnight at 4°C followed by 3 times washing with PBS. Secondary antibody incubation was performed for 1 h at room temperature and slides were washed thrice, 5 min each. Nuclei were counterstained with DAPI for 20 min at room temperature followed by washing with PBS. Excess of PBS was wiped out and mounting were performed using Prolog gold antifade reagent. The images were captured on confocal microscope (Olympus, Lambertville, NJ).

### ORF17 initial structure preparation and screening of natural compound database

PDB ID 4P3H was used to represent KSHV-encoded protease ORF17. Discovery studio 2.5 (DS, Accelrys Inc., San Diego) was used to remove all the water molecule except from the catalytic region. Addition of hydrogen atoms and optimization of side chain was performed using Prepwizard (Schrodinger suite). Prediction of Ionizable groups at neutral pH was performed by PROPKA. H-bond optimization was performed by ProtAssign. Impref utility module was used for structure minimization allowing restrain on heavy atoms only. The centroid of ligand N-[2-Benzyl-4-(1 h-Tetrazol-5-Yl) phenyl]-6-(Cyclohexylmethyl) pyridine-2-Carboxamide is used as the grid center. Chemical compounds of biological origin were downloaded from ZINC database. A library of 307,814 compounds of biological origin has been used as a screen library. Based on ionization states (ionization states possible between pH 5–9) and specified chirality (possible stereoisomer for specified carbon), the number of total compounds were 481,799. The list of top 10 ligands after the virtual screening is provided in Table 1.

**TABLE 1:** The top 10 ligands after the virtual screening. A library of 307,814 natural compounds of biological origin (a total 481,799 structures) has been used as a screen library.

ZINC_id	Conformer field	docking_score	glide_XP_GScore	glide_gscore	glide_ecoul	glide_energy	glide_einternal	glide_emodel
ZINC000261524046	Original	-11.112	-10.05795855	-10.057958	-12.49420	-44.685,596	11.42540455	-54.965,919
ZINC000261524046	MMFFS	-11.112	-10.05795855	-10.057958	-12.49420	-44.685,596	11.42540455	-54.965,919
ZINC000261524046	OPLS	-11.112	-10.05795855	-10.057958	-12.49420	-44.685,596	11.42540455	-54.965,919
ZINC000150349043	OPLS	-10.329	-9.940419012	-9.9404190	-9.188,562	-48.081220	10.73451424	-67.296,630
ZINC000100253766	Original	-10.058	-9.940419012	-9.9404190	-9.188,562	-48.081220	10.73451424	-67.296,630
ZINC000100253766	MMFFS	-10.058	-9.940419012	-9.9404190	-9.188,562	-48.081220	10.73451424	-67.296,630
ZINC000100253766	OPLS	-10.058	-9.896305987	-9.8963059	-11.86978	-35.563,805	3.140013695	-53.005601
ZINC000150349043	OPLS	-9.964	-9.896305987	-9.8963059	-11.86978	-35.563,805	3.140013695	-53.005601
ZINC000150349043	OPLS	-9.905	-9.896305987	-9.8963059	-11.86978	-35.563,805	3.140013695	-53.005601
ZINC000085511511	Original	-9.888	-9.976237585	-9.9762375	-3.4430971	-49.0701,379	14.30411148	-56.362,238

### Docking simulation, post-docking analysis and molecular dynamics simulation

Docking experiments were performed through a virtual screen workflow which combines glide module and high throughput virtual screening (Glide HTVS) with single precision (Glide SP) and extra precision (Glide XP). Extra precision provides high stringency by utilizing extensive conformational sampling. Prism MMGBSA was used to calculate the binding energy of protein-ligand complex derived from the Glide XP docking experiment. The VSGB2 was used as an implicit solvation model. The MMGBSA dG binding score was used to evaluate complexes using the formula dG bind = E_complex (minimized)-E_ligand (minimized)-E_protein (minimized). Desmond molecular dynamics and OPLS force field (version OPLS_2005) were used for molecular dynamics simulation of top scoring complexes. 10 Ǻ orthorhombic box was used as boundary conditions and TIP3P was used as solvation model for the molecular dynamics simulation. Neutralization of charges were made by adding Na^+^ ions. 0.15 M sodium chloride was added as salt. 200 ns time frame was used for simulation run under NPT conditions. Simulations were run at 300 K at 1.01 bars. The total number of atoms in the molecular system is 25,702. The heating and equilibration stages were done utilizing isothermal-isochoric ensemble (NVT) ad isothermal-isobaric ensemble (NPT) with restraints on solute heavy atoms using the desmond default settings. Nose-Hoover chain has been used as thermostat, whereas Martyna-Tobias-Klein has been sed as barostat. The relaxation time for thermostat and barostat was 1 ps and 2 ps, respectively.

### KSHV reactivation, inhibitor addition, viral preparation and copy number calculation

KSHV reactivation from naturally infected BC3 cells was performed as described earlier (Kumar Singh et al., 2021). Briefly, cells were grown at a concentration 5*10^5^ cells/ml in RPMI medium. Reactivation was initiated by adding TPA and butyric acid at a concentration of 5 μM/ml and 3 ng/ml respectively. For hypoxic reactivation, cells were grown under 1% oxygen condition for 72–96 h. ORF17 inhibitor ZINC00026154046 was added 12-h post reactivation with hypoxia or TPA/Butyric acid. Control and inhibitor containing cell culture flask were transferred to CO2 incubator and KSHV reactivation was allowed for 72-h. Cells along with medium were collected at the end of reactivation and centrifuged at 2000 rpm for 20 min to pellet down any cell debris. The clear supernatant was transferred into a 50 ml centrifuge tube, and the pellets were resuspended in 5 ml of PBS. The resuspended pellet was freeze-thawed thrice to break the cellular membrane and release intracellular KSHV virions, followed by centrifugation at 3,000 rpm for 30 min. All the supernatants were pooled together and filtered though 0.45 µM syringe filter and centrifuged at 25,000 rpm for 30 min to pellet down virions. Supernatant were aspirated and the pellets from different experiments were resuspended in equal volume of PBS. 200 µl of viral preparation was used for DNA isolation followed by copy number calculation. Briefly, equal volumes of 2X lysis buffer (20 mM Tris-pH 8.0, 2 mM EDTA and 300 mM NaCl) as added to viral preparation. SDS and proteinase K (final concentration 1% each) were added, and the samples were incubated at 55°C for 2 h. Standard phenol-chloroform extraction was used to isolate viral DNA. DNA pellet were dissolved in 200 µl DNase free water and 1 µl of DNA sample was used for KSHV copy number calculation as described earlier (Kumar Singh et al., 2021). KSHV copy number was calculated by the standard curve method using real-time PCR. Briefly, the known number of Cosmid construct containing KSHV genomic region (Co-ordinates: 85,820–100784) was used to generate a standard curve (30–300,000 copies in multiples of 10-fold). Genomic DNA isolated from purified KSHV virions from equal volumes of filtered supernatants were used to calculate the comparative number of KSHV generated in response of hypoxia or chemical chemically induced reactivation.

### Statistical analysis

Experiments were performed at least in triplicate. All the statistical analyses of this study were performed using freely available GraphPad Prism software (GraphPad, San Diego, CA). The mean values with standard error of mean were presented in this study when appropriate. Statistical significance in expression of different sets of experiment was calculated by performing a 2-tailed Student’s t test. The *p*-value of ≤0.05 was considered statistically significant. *, *p*-value < 0.05; ns denote non-significant change.

## Results

### Hypoxia and chemical inducers of KSHV reactivation up-regulates expression of KSHV-encoded protease (ORF17)

**FIGURE 1:**
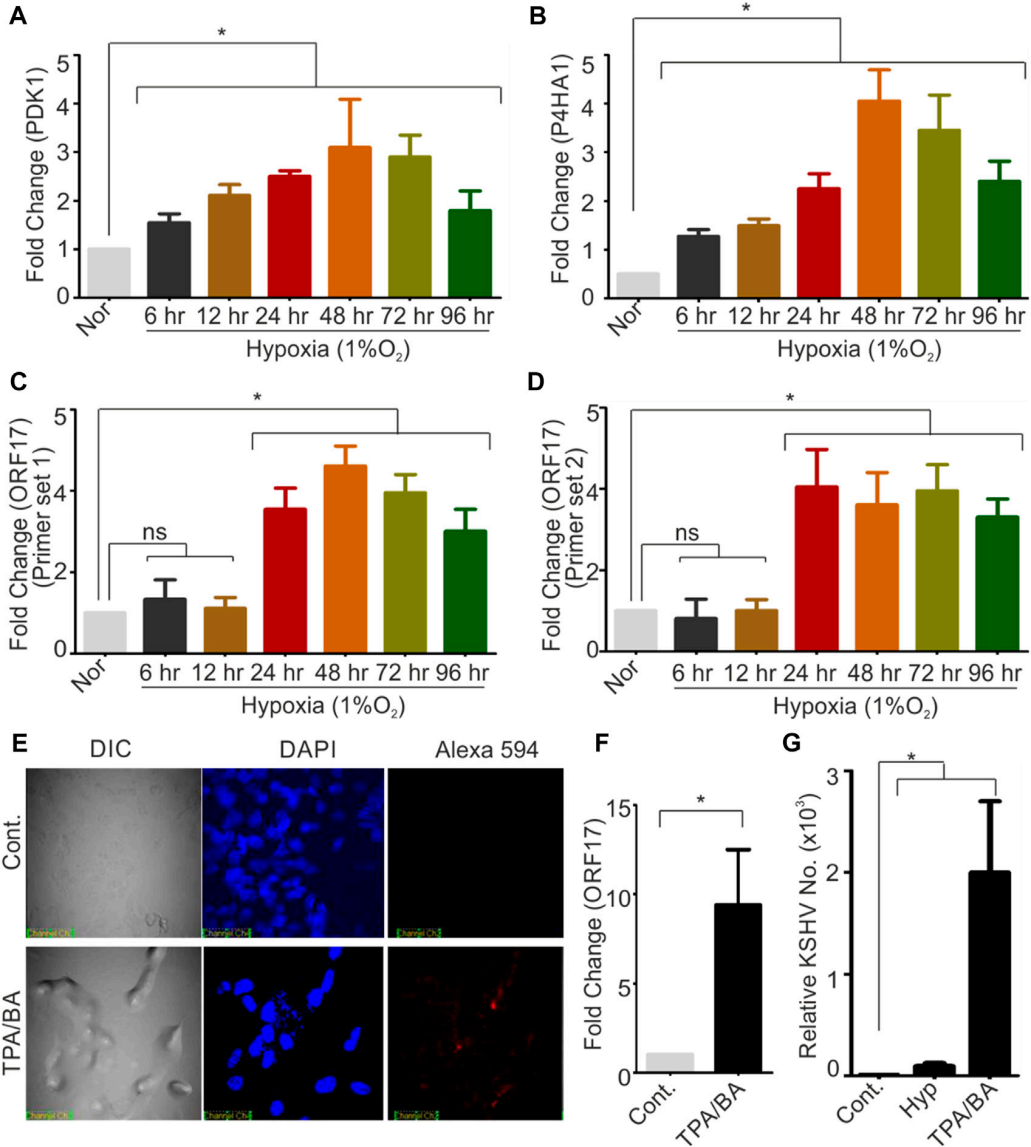
Hypoxia and chemical inducers of KSHV reactivation up-regulates expression of KSHV-encoded protease (ORF17). **(A,B)** BC3 cells were grown under normoxic or hypoxic conditions for indicated time periods. The induction of hypoxia was confirmed by analyzing real-time expression of hypoxic markers PDK1 and P4HA1. In brief, RNA from normoxic or hypoxic treated cells were isolated, and cDNA was synthesized. Equal amount of cDNA was used to monitor the expression by real-time PCR. GAPDH were used as internal control. **(C,D)** BC3 cells were grown under normoxic or hypoxic conditions for indicated time periods. Expression of KSHV-encoded protease (ORF17) were investigated by two different sets of primers as indicated. RNA from normoxic or hypoxic treated cells were isolated, and cDNA was synthesized. Equal amount of cDNA was used to monitor the expression by real-time PCR. GAPDH were used as internal control. **(E)** Reactivation of BC3 cells by treatment with TPA/Butyric acid was confirmed by immuno-staining with RTA antibodies. Nucleus were stained with DAPI and RTA were visuized by probing with secondary antibody conjugated with Alexa 594. **(F)** Real-time expression of ORF17 in control or TPA/BA reactivated BC3 cells. RNA was isolated from the control or TPA/BA reactivated BC3 cells. cDNA was synthesized and equal amount of cDNA was used to monitor the expression of ORF17 by real-time PCR. GAPDH were used as internal control. **(G)** Relative KSHV copy number calculation in control or hypoxia/chemically reactivated BC3 cells. Extracellular medium and cells (control or reactivated) were used to purify KSHV virions. Equal DNA from control or hypoxia/chemically reactivated BC3 cells were used to calculate the KSHV copy number. **(A–D and F–G)** All the experiments were performed in triplicate. Mean value with standard error of mean are shown. Asterisk represents statistically significant value with *p*-value less than or equal to 0.05 (ns = non-significant).

KSHV-encoded protease (ORF17 protein) plays an indispensable role during KSHV assembly and maturation during its reactivation and hence an up-regulated expression of KSHV-encoded ORF17 is anticipated phenomenon during KSHV reactivation. Since hypoxic reactivation of KSHV represents one of the physiological possible modes of KSHV reactivation, we hypothesized that hypoxia could up-regulate expression of KSHV-encoded ORF17. We performed a quantitative real-time PCR for investigating levels of ORF17 transcripts in KSHV positive cells grown under hypoxic conditions as compared to normoxic conditions. In brief, KSHV positive BC3 cells were grown in normoxic or hypoxic conditions for various time points followed by RNA isolation and cDNA synthesis. The confirmation of induction of hypoxia was made by investigating real-time expression of hypoxic markers such as PDK1 and P4HA1 (Figures 1A, B). A real-time PCR based method was used to determine the differential expression of ORF17 in the cells grown in hypoxic conditions as compared to their counterpart normoxic conditions. An up-regulation in the expression of ORF17 of up to approximately 5-fold (±1.5) at longer time periods was observed in BC3 cells (Figures 1C, D). Further, we wanted to confirm if the up-regulated expression ORF17 is a universal characteristic during KSHV reactivation and co-relate with viral production in reactivated conditions. We reactivated KSHV positive BC3 cells by treatment with butyric acid and TPA. The reactivation of BC3 cells was confirmed by immunofluorescence against KSHV-encoded RTA (Figure 1E). A real-time PCR based experiment to investigate expression profile of ORF17 in chemically reactivated BC3 cells showed that expression of ORF17 is even higher compared to the hypoxic reactivation (Figure 1F). We finally investigated if a direct co-relation between ORF17 expression and production of KSHV virions exists in reactivated condition. The results clearly indicated a proportional relationship between ORF17 expression and production of KSHV virions (Figure 1G). Altogether, these results confirmed that up-regulated expression of ORF17 is an associated event during hypoxic/chemically induced reactivation.

### Hypoxia inducible factor 1alpha (HIF1α) can directly up-regulate ORF17 expression

**FIGURE 2:**
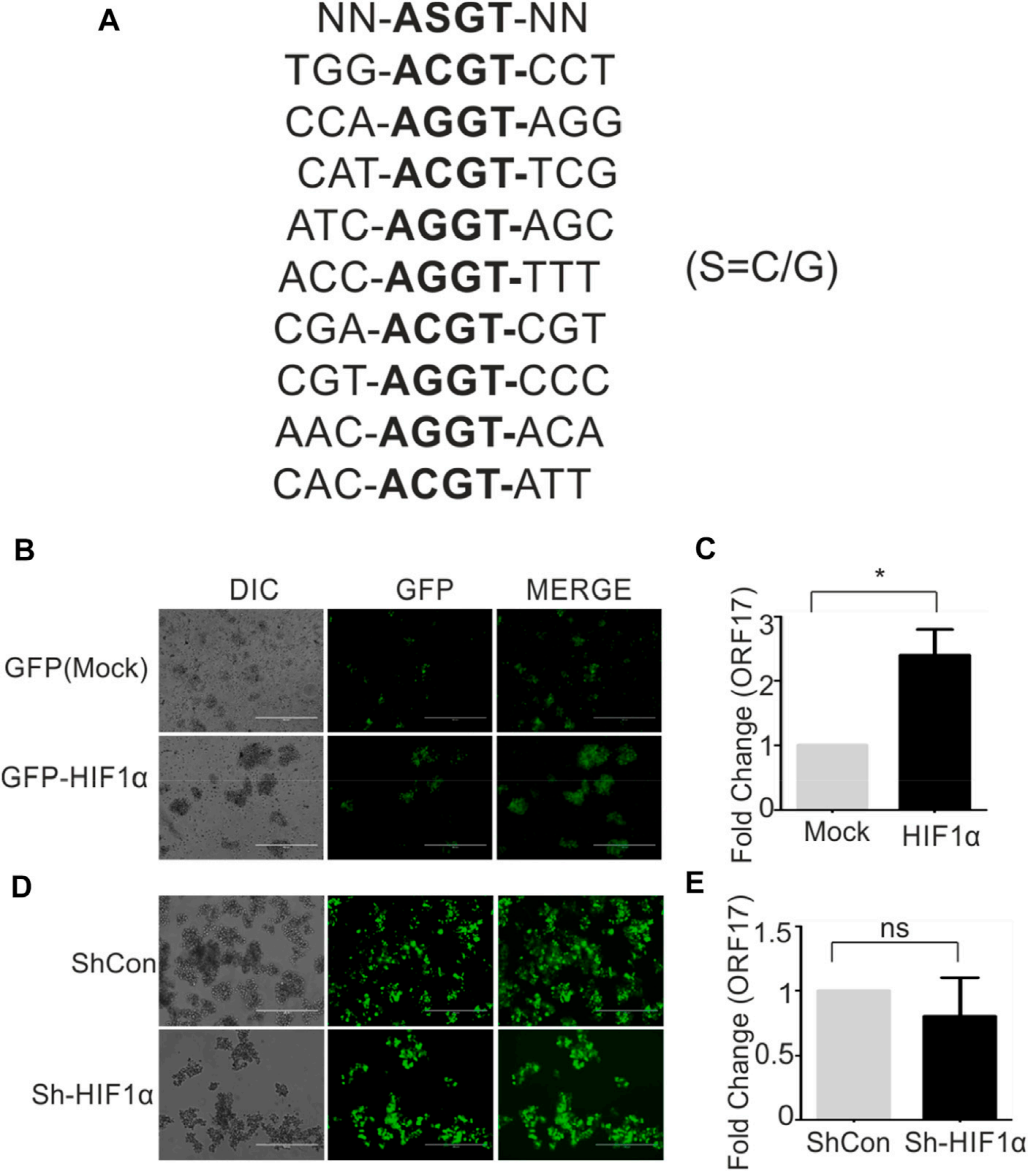
HIF1α can directly up-regulate expression of KSHV-encoded ORF17. **(A)** Presence of nine distinct hypoxia responsive elements (HREs) within 1,000 bp upstream of ORF17. NN-ASGT-NN represents HRE consensus sequence (S=C/G). **(B)** BC3 cells stably expressing Mock (GFP) or GFP-HIF1α. The expression was visualized by fluorescent microscopy. **(C)** A comparative study for real-time expression of ORF17 in mock or HIF1α expressing BC3 cells (* denotes *p* < 0.05). **(D)** BC3 cells stably expressing ShControl or ShHIF1α transcript. The expression was visualized by fluorescent microscopy. **(E)** A comparative study for real-time expression of ORF17 in expressing ShControl or ShHIF1α expressing BC3 cells (ns = non-significant).

HIF1α is a direct effector protein involved in hypoxic conditions. In hypoxic conditions, HIF1α stabilization occurs due to inactivation of vHL protein responsible for proteasomal degradation of HIF1α. In stabilized condition, HIF1α transactivate expression of several genes responsible for cell survival, metabolic reprogramming. These ensure hypoxic cells to withstand the negative impact of hypoxia till normoxic favorable conditions restores. The transactivation of genes by HIF1α depends on the presence of specific DNA consensus sequences [N-ASGT-NN (S=C/G)] called as hypoxia responsive elements. We screened 1,000 bp upstream sequence in the promoter region of ORF17 to find if any HRE is/are present in this sequence. Based on the presence of nine distinct HREs within the promoter region of ORF17 (Figure 2A), we investigated if ectopic expression of HIF1α can up-regulate expression of ORF17. Cells were transduced with either mock (GFP) or GFP-HIF1α expressing lentiviruses and successfully transduced cells were selected in puromycin containing medium. The expression of HIF1α was confirmed by fluorescence microscopy (Figure 2B). A comparative study for real-time expression of ORF17 in mock or HIF1α expressing cells showed that, ectopic expression of HIF1α can lead to up-regulated expression of ORF17 (Figure 2C). Although, the magnitude of elevated expression of ORF17 in this condition was lower as compared to cells grown under hypoxic conditions. To further corroborate these observations, we knocked down HIF1α in KSHV positive cells by ShRNA expressing lentiviruses. The successfully transduced cells were selected in puromycin containing medium and visualized by GFP fluorescence (Figure 2D). The cells were grown under either normoxic or hypoxic conditions followed by investigating expression of KSHV-encoded ORF17 in these cells. As expected, ORF17 expression was lower but statistically non-significant in ShHIF1α expressing cells (Figure 2E; *<0.05, ns = non-significant).

### Virtual screening and molecular docking of screened natural compound targeting KSHV-encoded protease (ORF17)

**FIGURE 3:**
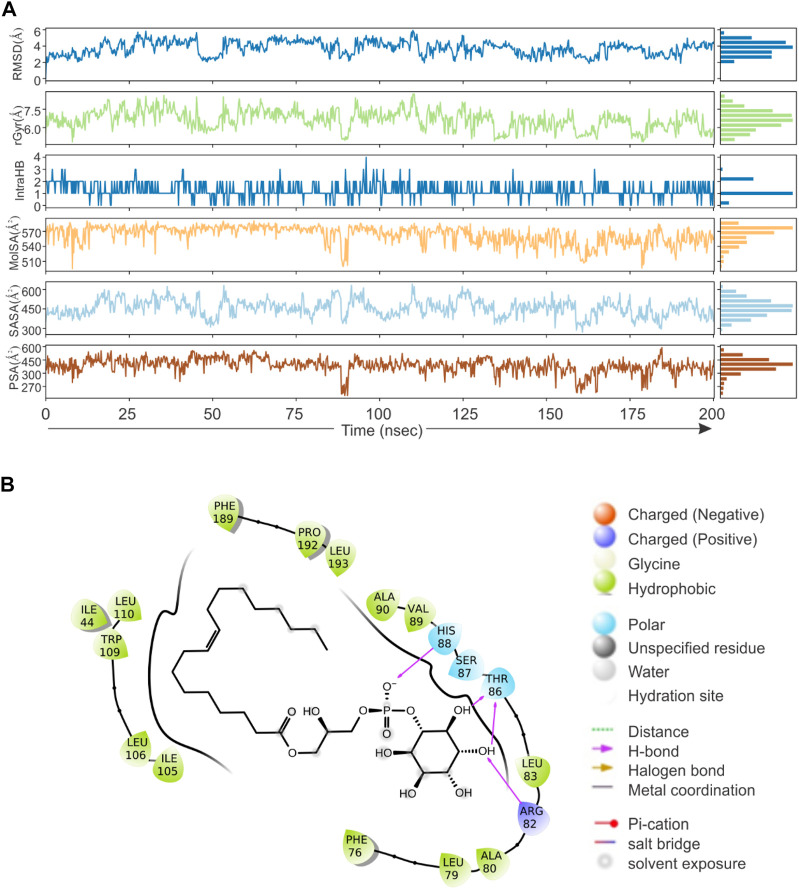
Virtual screening, docking and ligand property. A library of 307,814 compounds (a total 481,799 structure) has been used as a screen library. **(A)** RMSD, radius of gyration, MolSA, SASA and PSA characteristics of 1-oleoyl-2-hydroxy-sn-glycero-3-phospho-(1′-myo-inositol). **(B)** The molecular Interaction of 1-oleoyl-2-hydroxy-sn-glycero-3-phospho-(1′-myo-inositol) within active site of KSHV-encoded protease ORF17. Charged (positive/negative), glycine, hydrophobic, polar, water hydration sites, hydrogen bond, halogen bond, metal co-ordinates, Pi-cation, salt bridge and solvent exposure characteristics are shown.

**FIGURE 4:**
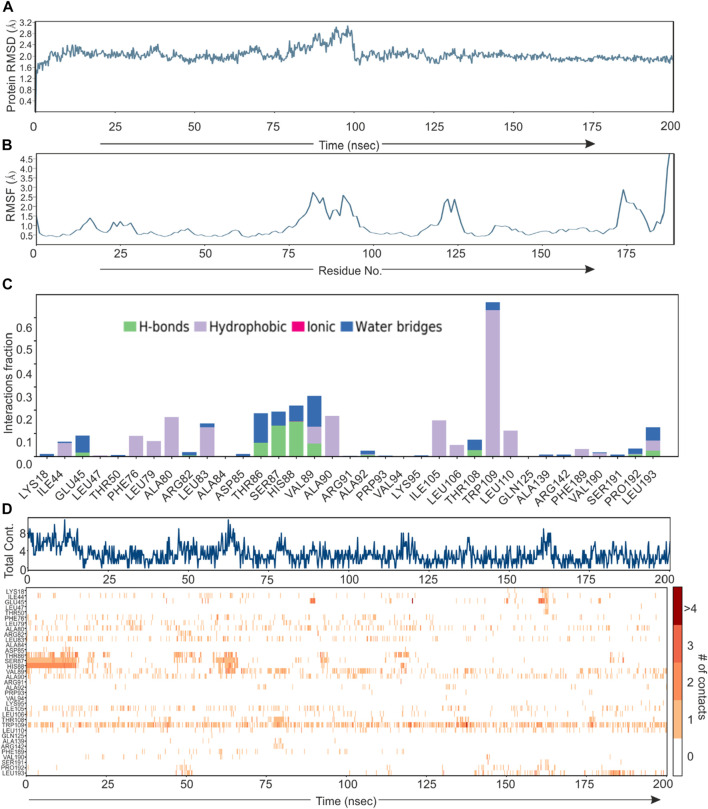
Root mean square deviation plot, Interaction plot and Interaction dynamics of KSHV-encoded protease and Lyso 18:1. **(A)** The KSHV-encoded protease is represented in blue color. *X*-axis represent the simulation time in nano second. **(B)** RMSF plot for KSHV-encoded protease. X-axis represents the residue number. **(C)** Interaction plot of KSHV-encoded protease with PLA. *Y*-axis represent the total number of contact fraction per residue per simulation time i.e. if the residue (presented on *X*-axis) has one contact during the whole simulation of 200 ns. **(D)** Interaction dynamics of KSHV-encoded protease with PLA. Glu 166 predominantly interacts with theaflavin digallate having contact varies from 2–4. The total number of contacts during simulation were ∼15.

Based on direct role of KSHV- encoded protease, we wanted to screen a natural product which can be utilized as its inhibitor for targeting KSHV-encoded protease and hence the reactivation. In a grid defined experimental setup, docking was performed as mentioned in materials and method section. A library of 307,814 natural compounds of biological origin has been used as a screen library. Based on ionization states (ionization states possible between pH 5–9) and specified chirality (possible stereoisomer for specified carbon), the number of total compounds were 481,799. Several parameters based on scoring functions, such as docking score, energy score, XP Gscore, glide gscore, were used to identify aa dominant inhibitor of KSHV-encoded protease ORF17. Based on the docking score, top 10 compounds are shown in Table 1. ZINC000261524046 (original conformer; 1-oleoyl-2-hydroxy-sn-glycero-3-phospho-(1′-myo-inositol was selected for further studies based on its highest docking score. 1-oleoyl-2-hydroxy-sn-glycero-3-phospho-(1′-myo-inositol), is an easily available lysophosphatidic acid and showed a high binding and hence inhibitory characteristics. A RMSD, radius of gyration, MolSA, SASA and PSA study was performed to investigate ligand properties (Figure 3A). The molecular Interaction of ligand with protease is shown in Figure 3B. Briefly, the bonding and characteristics such as charged (positive/negative), glycine, hydrophobic, polar, water hydration sites, hydrogen bond, halogen bond, metal co-ordinates, Pi-cation, salt bridge and solvent exposure were utilized (Figure 3B). The main hydrogen bond at His88, Thr86 and Arg82 were identified (Figure 3B). We further performed protein RMSD, protein RMSF, interaction fraction and protein contact studies. A protein RMSD plot for 200 nsec is shown in Figure 4A. RMSF values till 200 proteins residues in shown in Figure 4B. The interaction fraction of important residues in the terms of hydrogen bonding, hydrophobic bonding, ionic bonding and water bridge is shown in Figure 4C. The important hydrophobic bonding regions were concentrated near PHE76, LEU79, ALA80, ALA90 and THR109. Similarly, hydrogen bonding’s were maximum observed for THR86, SER87, HIS88 and VAL89 (Figure 4C). A timeline representation for contact and interactions are shown in Figure 4D.

### LPA inhibits KSHV production from the reactivated BC3 cells

**FIGURE 5:**
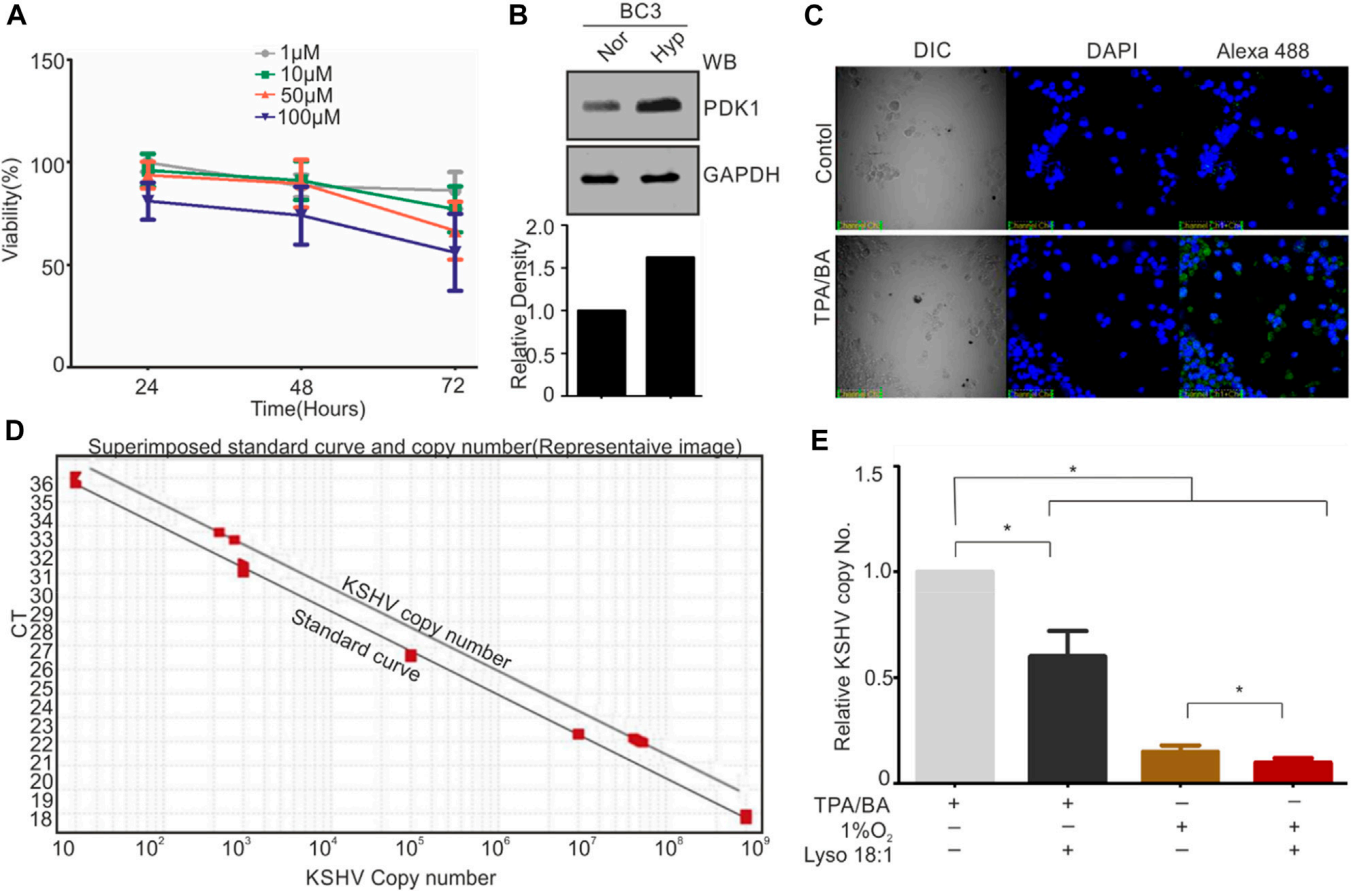
LPA inhibits KSHV production of KSHV virions from reactivated cells. **(A)** Cell viability test in different concentration of LPA. Cells were grown in presence of different concentration of LPA for the indicated time periods. Viability of cells was checked by Trypan blue staining. Mean value of viable cells from three independent experiments are shown. **(B)** Induction of hypoxia during hypoxic reactivation was confirmed by PDK1 Western blot. GAPDH served as loading control. **(C)** TPA/BA mediated reactivation of BC3 cells was confirmed by RTA immunofluorescence. **(D)** Standard curve for various KSHV copy number plasmids superimposed with curve for KSHV copy number calculation. **(E)** Relative KSHV copy number from extracellular medium of TPA/BA or hypoxia reactivated cells as compared with or without LPA treatment.

Based on the *in-vitro* results showing inhibitory effect of KSHV-encoded protease by LPA, we wanted to validate this result in cell culture-based experiments. Frist, the cytotoxicity of LPA on KSHV-positive BC3 cells was investigated by treating these cells with increasing concentration (1–100 µM) for 24–72 h (Figure 5A). The 1 μM and 10 µM concentration showed minimal cytotoxic effect on BC3 cells, with retained viability of 95 ± 3% at 24 h and 85 ± 5% at 72 h. However, 100 µM concentration of LPA showed approximately 85 ± 5% even at 24 h of treatment. Treatment of these cells with 50 µM of LPA showed an intermediate effect on cell viability (Figure 5A). Based on the viability test results, we choose to perform further experiments using 1 µM concentration of LPA. BC3 cells were reactivated by either growing under hypoxic conditions (1%O_2_) or by adding TPA/BA in the growing culture. Induction of Hypoxia was confirmed by PDK1 Western blot (Figure 5B). KSHV reactivation in response to TPA/BA was confirmed by immunofluorescence against KSHV-encoded RTA (Figure 5C). 12-h post reactivation, LPA at a final concentration of 1 µM was added to the cells. For KSHV copy number calculation from extra cellular medium of reactivated cells, a standard curve with known number of KSHV genomic region cloned in cosmid vector was generated (Figure 5D). In brief, clone of KSHV genomic region encompassing 85,000-102000 bp in cosmid was serially diluted to obtained 30 to 30^5^ copies of cloned region per µl of solution. 1µl sample from each of the dilution was used to generate a standard curve for the known number of KSHV specific DNA. The extra-cellular medium from the reactivated cells were collected and excreted KSHV virions were collected by centrifugation followed by isolation of viral DNA. Equal DNA was used for copy number calculation. The superimposed image for the standard curve and the copy number calculation is showed in Figure 5D. The results showed a statistically significant reduction in the production of KSHV virions in the cells treated with LPA. Interestingly, the results also suggested that hypoxic reactivation is very less efficient as compared to TPA/BA mediated reactivation. Nevertheless, LPA inhibited production of KSHV virions in hypoxia reactivated cells in the same manner as that of TPA/BA activated cells.

## Discussion

KSHV-associated malignancies represent a great challenge in immuno-compromised individuals and highly prevalent in HIV-infected individuals or among the individuals with organ transplants undergoing immunosuppressive medications (Ganem, 2010; Cesarman et al., 2019). Several KSHV-encoded antigens are known to participate in KSHV associated pathogenesis (Cavallin et al., 2014). LANA has received high attention within groups working in the field of KSHV biology. LANA is considered as master regulator in KSHV pathogenesis based on its indispensable role in maintenance of latency, KSHV replication in latently infected cells and manipulation of cell cycle & DNA replication pathways by interacting or degrading components of these pathways (Uppal et al., 2014; Purushothaman et al., 2016; Wei et al., 2016). As a therapeutics approach several groups are working to develop strategies to target LANA (Calderon et al., 2020). The other KSHV-encoded protein which remains a central focus for the development of anti-KSHV strategy is KSHV-encoded DNA polymerase (Coen et al., 2014). KSHV-encoded DNA polymerase plays important role in lytic replication of virus, while latent replication is supported by host polymerase (Purushothaman et al., 2015). Targeting KSHV-encoded DNA polymerase is an anticipated strategy for dissemination of viral particle upon reactivation challenges (Purushothaman et al., 2015). Nevertheless, other antigens encoded by KSHV are equivocally claimed for the KSHV pathogenesis (Mariggio et al., 2017). KSHV-encode vGPCR promotes cellular transformation by acting on MAPK-P38 pathway to activate HIF1α transcription and associated pathways (Sodhi et al., 2000). KSHV-encoded vCyclin directly influence cell cycle progression by overriding contact inhibition (Jones et al., 2014). KSHV-encoded vCyclin also interacts with several cyclin dependent kinases including CDK6 to phosphorylate several proteins such as histones, retinoblastoma, origin recognition proteins (ORCs), bcl-2, p53 etc (Sarek et al., 2010). These generally lead to cell cycle progression and eventually cellular transformation (Chang et al., 1996; Godden-Kent et al., 1997). KSHV-encoded vFLIP, interferon regulatory factors (vIRFs) and micro RNAs are also involved in cellular transformation by regulating various cellular pathways (An et al., 2003; Jacobs and Damania, 2011; Qin et al., 2012; Hwang et al., 2017; Qin et al., 2017). Based on these multiple modes for the pathogenesis, treatment of KSHV related malignancies remain a challenge to the scientific community (Arav-Boger, 2009; Moore and Chang, 2011). In many cases, antiviral therapy has been suggested as an effective approach for the management and treatment of KSHV associated pathological conditions (Arav-Boger, 2009; Lurain et al., 2018).

Like many other viruses, KSHV also encodes a protease with high importance in assembly and maturation of viral particles. Viral encoded proteases are essential for assembly and packaging of progeny viruses and are generally expressed during the viral reactivation (Veesler and Johnson, 2012). In the present study, we investigated expression profile of KSHV-encoded protease and its relevance in the viral reactivation during physiological allowed condition of hypoxia and compared with chemically induced reactivation. Although, hypoxic reactivation of KSHV is a well-established phenomenon, a long-term hypoxic treatment is required for generating enough viral progenies (Singh et al., 2019; Kumar Singh et al., 2021). HIF1α, the key transcription factor and major player under hypoxic conditions, does not represent a good marker for showing hypoxic induction in long term hypoxic treatment (Uchida et al., 2004). We used expression levels of PDK1 and P4HA1 to demonstrate induction of hypoxia. Analysis for the expression pattern for the KSHV-encoded protease showed a proportional relationship between its expression and the time of hypoxic induction. The expression of KSHV-encoded protease was also higher in chemically reactivated cells. These results suggested a key role of KSHV-encoded protease in KSHV reactivation (Figure 1). Interestingly, ectopic expression of HIF1α was also sufficient to induce expression of KSHV-encoded protease. HIF1α is already reported to transactivate expression of viral transcription and replication activator (RTA). Up-regulated expression of KSHV protease in hypoxia or chemically reactivated cells suggested a dual mode of its transactivation, where HIF1α alone or through RTA can transactivate expression of KSHV-encoded protease (Figure 2). Virtual screening of natural products was performed to identify a easily accessible molecule which can target and inhibit KSHV-encoded protease. Based on the docking score of the top scoring compounds (Table two), 1-oleoyl-2-hydroxy-sn-glycero-3-phospho-(1′-myo-inositol) was chosen for *in-vitro* molecular dynamics experiments. A reliable and encouraging result of *in-vitro* experiment allowed us to investigate effect of this molecule in cell culture-based experiments. The cytotoxicity of the compound was investigated by viability test using trypan blue staining. The compound appeared very mild toxic, especially at the lower concentration (1–10 µM). KSHV-positive BC3 cells reactivated followed by treatment with 1-oleoyl-2-hydroxy-sn-glycero-3-phospho-(1′-myo-inositol) to investigate its effect on viral production. The results were very encouraging and showed a clear inhibition in the production of mature virions under hypoxic or chemically induced reactivation. Overall, the study propose use of 1-oleoyl-2-hydroxy-sn-glycero-3-phospho-(1′-myo-inositol) as an alternate approach in management of KSHV associated pathogenesis.

## Data Availability

The original contributions presented in the study are included in the article/supplementary material, further inquiries can be directed to the corresponding author.

## References

[B1] Alomari N. Totonchy J. (2020). Cytokine-Targeted therapeutics for KSHV-associated disease. Viruses 12 (10), 1097. PubMed PMID: 32998419; PubMed Central PMCID: PMCPMC7600567. 10.3390/v12101097 32998419 PMC7600567

[B2] An J. Sun Y. Sun R. Rettig M. B. (2003). Kaposi's sarcoma-associated herpesvirus encoded vFLIP induces cellular IL-6 expression: The role of the NF-kappaB and JNK/AP1 pathways. Oncogene 22 (22), 3371–3385. PubMed PMID: 12776188. 10.1038/sj.onc.1206407 12776188

[B3] Aneja K. K. Yuan Y. (2017). Reactivation and lytic replication of kaposi's sarcoma-associated herpesvirus: An update. Front. Microbiol. 8, 613. PubMed PMID: 28473805; PubMed Central PMCID: PMCPMC5397509. 10.3389/fmicb.2017.00613 28473805 PMC5397509

[B4] Antman K. Chang Y. (2000). Kaposi's sarcoma. N. Engl. J. Med. 342 (14), 1027–1038. PubMed PMID: 10749966. 10.1056/NEJM200004063421407 10749966

[B5] Arav-Boger R. (2009). Treatment for Kaposi sarcoma herpesvirus: Great challenges with promising accomplishments. Virus Genes 38 (2), 195–203. PubMed PMID: 19139983. 10.1007/s11262-008-0325-y 19139983

[B6] Ballestas M. E. Kaye K. M. (2011). The latency-associated nuclear antigen, a multifunctional protein central to Kaposi's sarcoma-associated herpesvirus latency. Future Microbiol. 6 (12), 1399–1413. PubMed PMID: 22122438; PubMed Central PMCID: PMCPMC3857968. 10.2217/fmb.11.137 22122438 PMC3857968

[B7] Bhutani M. Polizzotto M. N. Uldrick T. S. Yarchoan R. (2015). Kaposi sarcoma-associated herpesvirus-associated malignancies: Epidemiology, pathogenesis, and advances in treatment. Semin. Oncol. 42 (2), 223–246. PubMed PMID: 25843728; PubMed Central PMCID: PMCPMC6309362. 10.1053/j.seminoncol.2014.12.027 25843728 PMC6309362

[B8] Butnaru M. Gaglia M. M. (2018). Transcriptional and post-transcriptional regulation of viral gene expression in the gamma-herpesvirus Kaposi's sarcoma-associated herpesvirus. Curr. Clin. Microbiol. Rep. 5 (4), 219–228. PubMed PMID: 30854283; PubMed Central PMCID: PMCPMC6405233. 10.1007/s40588-018-0102-1 30854283 PMC6405233

[B9] Cai Q. L. Knight J. S. Verma S. C. Zald P. Robertson E. S. (2006). EC5S ubiquitin complex is recruited by KSHV latent antigen LANA for degradation of the VHL and p53 tumor suppressors. PLoS Pathog. 2 (10), e116. PubMed PMID: 17069461; PubMed Central PMCID: PMCPMC1626105. 10.1371/journal.ppat.0020116 17069461 PMC1626105

[B10] Calderon A. Soldan S. S. De Leo A. Deng Z. Frase D. M. Anderson E. M. (2020). Identification of Mubritinib (TAK 165) as an inhibitor of KSHV driven primary effusion lymphoma via disruption of mitochondrial OXPHOS metabolism. Oncotarget 11 (46), 4224–4242. PubMed PMID: 33245718; PubMed Central PMCID: PMCPMC7679036. 10.18632/oncotarget.27815 33245718 PMC7679036

[B11] Campbell M. Izumiya Y. (2012). Post-translational modifications of kaposi's sarcoma-associated herpesvirus regulatory proteins - SUMO and KSHV. Front. Microbiol. 3, 31. PubMed PMID: 22347876; PubMed Central PMCID: PMCPMC3278983. 10.3389/fmicb.2012.00031 22347876 PMC3278983

[B12] Campbell M. Yang W. S. Yeh W. W. Kao C. H. Chang P. C. (2020). Epigenetic regulation of kaposi's sarcoma-associated herpesvirus latency. Front. Microbiol. 11, 850. PubMed PMID: 32508765; PubMed Central PMCID: PMCPMC7248258. 10.3389/fmicb.2020.00850 32508765 PMC7248258

[B13] Cavallin L. E. Goldschmidt-Clermont P. Mesri E. A. (2014). Molecular and cellular mechanisms of KSHV oncogenesis of Kaposi's sarcoma associated with HIV/AIDS. PLoS Pathog. 10 (7), e1004154. PubMed PMID: 25010730; PubMed Central PMCID: PMCPMC4092131. 10.1371/journal.ppat.1004154 25010730 PMC4092131

[B14] Cesarman E. Damania B. Krown S. E. Martin J. Bower M. Whitby D. (2019). Kaposi sarcoma. Nat. Rev. Dis. Prim. 5 (1), 9. PubMed PMID: 30705286; PubMed Central PMCID: PMCPMC6685213. 10.1038/s41572-019-0060-9 30705286 PMC6685213

[B15] Chang Y. Cesarman E. Pessin M. S. Lee F. Culpepper J. Knowles D. M. (1994). Identification of herpesvirus-like DNA sequences in AIDS-associated Kaposi's sarcoma. Science 266 (5192), 1865–1869. PubMed PMID: 7997879. 10.1126/science.7997879 7997879

[B16] Chang Y. Moore P. S. Talbot S. J. Boshoff C. H. Zarkowska T. Godden K. (1996). Cyclin encoded by KS herpesvirus. Nature 382 (6590), 410. PubMed PMID: 8684480. 10.1038/382410a0 8684480

[B17] Chen J. Dai L. Goldstein A. Zhang H. Tang W. Forrest J. C. (2019). Identification of new antiviral agents against Kaposi's sarcoma-associated herpesvirus (KSHV) by high-throughput drug screening reveals the role of histamine-related signaling in promoting viral lytic reactivation. PLoS Pathog. 15 (12), e1008156. PubMed PMID: 31790497; PubMed Central PMCID: PMCPMC6907871. 10.1371/journal.ppat.1008156 31790497 PMC6907871

[B18] Coen N. Duraffour S. Snoeck R. Andrei G. (2014). KSHV targeted therapy: An update on inhibitors of viral lytic replication. Viruses 6 (11), 4731–4759. PubMed PMID: 25421895; PubMed Central PMCID: PMCPMC4246246. 10.3390/v6114731 25421895 PMC4246246

[B19] Ganem D. (2010). KSHV and the pathogenesis of Kaposi sarcoma: Listening to human biology and medicine. J. Clin. Invest. 120 (4), 939–949. PubMed PMID: 20364091; PubMed Central PMCID: PMCPMC2847423. 10.1172/JCI40567 20364091 PMC2847423

[B20] Godden-Kent D. Talbot S. J. Boshoff C. Chang Y. Moore P. Weiss R. A. (1997). The cyclin encoded by Kaposi's sarcoma-associated herpesvirus stimulates cdk6 to phosphorylate the retinoblastoma protein and histone H1. J. Virol. 71 (6), 4193–4198. PubMed PMID: 9151805; PubMed Central PMCID: PMCPMC191633. 10.1128/JVI.71.6.4193-4198.1997 9151805 PMC191633

[B21] Goldsmith C. S. Miller S. E. (2009). Modern uses of electron microscopy for detection of viruses. Clin. Microbiol. Rev. 22 (4), 552–563. PubMed PMID: 19822888; PubMed Central PMCID: PMCPMC2772359. 10.1128/CMR.00027-09 19822888 PMC2772359

[B22] Gunther T. Grundhoff A. (2010). The epigenetic landscape of latent Kaposi sarcoma-associated herpesvirus genomes. PLoS Pathog. 6 (6), e1000935. PubMed PMID: 20532208; PubMed Central PMCID: PMCPMC2880564. 10.1371/journal.ppat.1000935 20532208 PMC2880564

[B23] Hume A. J. Kalejta R. F. (2009). Regulation of the retinoblastoma proteins by the human herpesviruses. Cell Div. 4, 1. PubMed PMID: 19146698; PubMed Central PMCID: PMCPMC2636798. 10.1186/1747-1028-4-1 19146698 PMC2636798

[B24] Hwang S. W. Kim D. Jung J. U. Lee H. R. (2017). KSHV-encoded viral interferon regulatory factor 4 (vIRF4) interacts with IRF7 and inhibits interferon alpha production. Biochem. Biophys. Res. Commun. 486 (3), 700–705. PubMed PMID: 28342865; PubMed Central PMCID: PMCPMC5490377. 10.1016/j.bbrc.2017.03.101 28342865 PMC5490377

[B25] Jacobs S. R. Damania B. (2011). The viral interferon regulatory factors of KSHV: Immunosuppressors or oncogenes? Front. Immunol. 2, 19. PubMed PMID: 22566809; PubMed Central PMCID: PMCPMC3342017. 10.3389/fimmu.2011.00019 22566809 PMC3342017

[B26] Jenner R. G. Alba M. M. Boshoff C. Kellam P. (2001). Kaposi's sarcoma-associated herpesvirus latent and lytic gene expression as revealed by DNA arrays. J. Virol. 75 (2), 891–902. PubMed PMID: 11134302; PubMed Central PMCID: PMCPMC113985. 10.1128/JVI.75.2.891-902.2001 11134302 PMC113985

[B27] Jones T. Ramos da Silva S. Bedolla R. Ye F. Zhou F. Gao S. J. (2014). Viral cyclin promotes KSHV-induced cellular transformation and tumorigenesis by overriding contact inhibition. Cell Cycle 13 (5), 845–858. PubMed PMID: 24419204; PubMed Central PMCID: PMCPMC3979920. 10.4161/cc.27758 24419204 PMC3979920

[B28] Juillard F. Tan M. Li S. Kaye K. M. (2016). Kaposi's sarcoma herpesvirus genome persistence. Front. Microbiol. 7, 1149. PubMed PMID: 27570517; PubMed Central PMCID: PMCPMC4982378. 10.3389/fmicb.2016.01149 27570517 PMC4982378

[B29] Karass M. Grossniklaus E. Seoud T. Jain S. Goldstein D. A. (2017). Kaposi sarcoma inflammatory cytokine syndrome (KICS): A rare but potentially treatable condition. Oncologist 22 (5), 623–625. PubMed PMID: 28424322; PubMed Central PMCID: PMCPMC5423516 article. 10.1634/theoncologist.2016-0237 28424322 PMC5423516

[B30] Kumar Singh R. Pei Y. Bose D. Lamplugh Z. L. Sun K. Yuan Y. (2021). KSHV-encoded vCyclin can modulate HIF1α levels to promote DNA replication in hypoxia. Elife 10, e57436. PubMed PMID: 34279223; PubMed Central PMCID: PMCPMC8315796. 10.7554/eLife.57436 34279223 PMC8315796

[B31] Lu F. Tsai K. Chen H. S. Wikramasinghe P. Davuluri R. V. Showe L. (2012). Identification of host-chromosome binding sites and candidate gene targets for Kaposi's sarcoma-associated herpesvirus LANA. J. Virol. 86 (10), 5752–5762. PubMed PMID: 22419807; PubMed Central PMCID: PMCPMC3347294. 10.1128/JVI.07216-11 22419807 PMC3347294

[B32] Lurain K. Yarchoan R. Uldrick T. S. (2018). Treatment of Kaposi sarcoma herpesvirus-associated multicentric castleman disease. Hematol. Oncol. Clin. North Am. 32 (1), 75–88. PubMed PMID: 29157621; PubMed Central PMCID: PMCPMC5726416. 10.1016/j.hoc.2017.09.007 29157621 PMC5726416

[B33] Mariggio G. Koch S. Schulz T. F. (2017). Kaposi sarcoma herpesvirus pathogenesis. Philos. Trans. R. Soc. Lond B Biol. Sci. 372 (1732), 20160275. PubMed PMID: 28893942; PubMed Central PMCID: PMCPMC5597742. 10.1098/rstb.2016.0275 28893942 PMC5597742

[B34] Mlombe Y. (2008). Management of HIV associated Kaposi's sarcoma in Malawi. Malawi Med. J. 20 (4), 129–132. PubMed PMID: 19537395; PubMed Central PMCID: PMCPMC3345707. 10.4314/mmj.v20i4.10975 19537395 PMC3345707

[B35] Moore P. S. Chang Y. (1995). Detection of herpesvirus-like DNA sequences in Kaposi's sarcoma in patients with and those without HIV infection. N. Engl. J. Med. 332 (18), 1181–1185. PubMed PMID: 7700310. 10.1056/NEJM199505043321801 7700310

[B36] Moore P. S. Chang Y. (2011). KSHV: Forgotten but not gone. Blood 117 (26), 6973–6974. PubMed PMID: 21719604. 10.1182/blood-2011-05-350306 21719604

[B37] Munz C. (2018). Human gamma-herpesvirus infection, tumorigenesis, and immune control in mice with reconstituted human immune system components. Front. Immunol. 9, 238. PubMed PMID: 29483919; PubMed Central PMCID: PMCPMC5816265. 10.3389/fimmu.2018.00238 29483919 PMC5816265

[B38] Narkhede M. Arora S. Ujjani C. (2018). Primary effusion lymphoma: Current perspectives. Onco Targets Ther. 11, 3747–3754. PubMed PMID: 29988764; PubMed Central PMCID: PMCPMC6029609. 10.2147/OTT.S167392 29988764 PMC6029609

[B39] Parravicini C. Chandran B. Corbellino M. Berti E. Paulli M. Moore P. S. (2000). Differential viral protein expression in Kaposi's sarcoma-associated herpesvirus-infected diseases: Kaposi's sarcoma, primary effusion lymphoma, and multicentric Castleman's disease. Am. J. Pathol. 156 (3), 743–749. PubMed PMID: 10702388; PubMed Central PMCID: PMCPMC1876837. 10.1016/S0002-9440(10)64940-1 10702388 PMC1876837

[B40] Purushothaman P. Dabral P. Gupta N. Sarkar R. Verma S. C. (2016). KSHV genome replication and maintenance. Front. Microbiol. 7, 54. PubMed PMID: 26870016; PubMed Central PMCID: PMCPMC4740845. 10.3389/fmicb.2016.00054 26870016 PMC4740845

[B41] Purushothaman P. Uppal T. Verma S. C. (2015). Molecular biology of KSHV lytic reactivation. Viruses 7 (1), 116–153. PubMed PMID: 25594835; PubMed Central PMCID: PMCPMC4306831. 10.3390/v7010116 25594835 PMC4306831

[B42] Qin J. Li W. Gao S. J. Lu C. (2017). KSHV microRNAs: Tricks of the devil. Trends Microbiol. 25 (8), 648–661. PubMed PMID: 28259385; PubMed Central PMCID: PMCPMC6904892. 10.1016/j.tim.2017.02.002 28259385 PMC6904892

[B43] Qin Z. Jakymiw A. Findlay V. Parsons C. (2012). KSHV-encoded MicroRNAs: Lessons for viral cancer pathogenesis and emerging concepts. Int. J. Cell Biol. 2012, 603961. PubMed PMID: 22505930; PubMed Central PMCID: PMCPMC3296157. 10.1155/2012/603961 22505930 PMC3296157

[B44] Raeisi D. Payandeh M. Madani S. H. Zare M. E. Kansestani A. N. Hashemian A. H. (2013). Kaposi's sarcoma after kidney transplantation: A 21-years experience. Int. J. Hematol. Oncol. Stem Cell Res. 7 (4), 29–33. PubMed PMID: 24505540; PubMed Central PMCID: PMCPMC3915423. 24505540 PMC3915423

[B45] Rihana N. Nanjappa S. Sullivan C. Velez A. P. Tienchai N. Greene J. N. (2018). Malignancy trends in HIV-infected patients over the past 10 Years in a single-center retrospective observational study in the United States. Cancer . 25 (1), 1073274818797955. PubMed PMID: 30185062; PubMed Central PMCID: PMCPMC6128080. 10.1177/1073274818797955 PMC6128080 30185062

[B46] Sarek G. Jarviluoma A. Moore H. M. Tojkander S. Vartia S. Biberfeld P. (2010). Nucleophosmin phosphorylation by v-cyclin-CDK6 controls KSHV latency. PLoS Pathog. 6 (3), e1000818. PubMed PMID: 20333249; PubMed Central PMCID: PMCPMC2841626. 10.1371/journal.ppat.1000818 20333249 PMC2841626

[B47] Singh R. K. Lamplugh Z. L. Lang F. Yuan Y. Lieberman P. You J. (2019). KSHV-encoded LANA protects the cellular replication machinery from hypoxia induced degradation. PLoS Pathog. 15 (9), e1008025. PubMed PMID: 31479497; PubMed Central PMCID: PMCPMC6743784. 10.1371/journal.ppat.1008025 31479497 PMC6743784

[B48] Singh R. K. Lang F. Pei Y. Jha H. C. Robertson E. S. (2018). Metabolic reprogramming of Kaposi's sarcoma associated herpes virus infected B-cells in hypoxia. PLoS Pathog. 14 (5), e1007062. PubMed PMID: 29746587; PubMed Central PMCID: PMCPMC5963815. 10.1371/journal.ppat.1007062 29746587 PMC5963815

[B49] Sissolak G. Mayaud P. (2005). AIDS-Related kaposi's sarcoma: Epidemiological, diagnostic, treatment and control aspects in sub-saharan africa. Trop. Med. Int. Health 10 (10), 981–992. PubMed PMID: 16185232. 10.1111/j.1365-3156.2005.01491.x 16185232

[B50] Sodhi A. Montaner S. Patel V. Zohar M. Bais C. Mesri E. A. (2000). The Kaposi's sarcoma-associated herpes virus G protein-coupled receptor up-regulates vascular endothelial growth factor expression and secretion through mitogen-activated protein kinase and p38 pathways acting on hypoxia-inducible factor 1alpha. Cancer Res. 60 (17), 4873–4880. PubMed PMID: 10987301. 10987301

[B51] Staudt M. R. Dittmer D. P. (2007). The Rta/Orf50 transactivator proteins of the gamma-herpesviridae. Curr. Top. Microbiol. Immunol. 312, 71–100. PubMed PMID: 17089794. 10.1007/978-3-540-34344-8_3 17089794

[B52] Sullivan R. J. Pantanowitz L. Dezube B. J. (2009). Targeted therapy for Kaposi sarcoma. BioDrugs 23 (2), 69–75. PubMed PMID: 19489649; PubMed Central PMCID: PMCPMC2707492. 10.2165/00063030-200923020-00001 19489649 PMC2707492

[B53] Toth Z. Maglinte D. T. Lee S. H. Lee H. R. Wong L. Y. Brulois K. F. (2010). Epigenetic analysis of KSHV latent and lytic genomes. PLoS Pathog. 6 (7), e1001013. PubMed PMID: 20661424; PubMed Central PMCID: PMCPMC2908616. 10.1371/journal.ppat.1001013 20661424 PMC2908616

[B54] Uchida T. Rossignol F. Matthay M. A. Mounier R. Couette S. Clottes E. (2004). Prolonged hypoxia differentially regulates hypoxia-inducible factor (HIF)-1alpha and HIF-2alpha expression in lung epithelial cells: Implication of natural antisense HIF-1alpha. J. Biol. Chem. 279 (15), 14871–14878. PubMed PMID: 14744852. 10.1074/jbc.M400461200 14744852

[B55] Uldrick T. S. Polizzotto M. N. Yarchoan R. (2012). Recent advances in Kaposi sarcoma herpesvirus-associated multicentric Castleman disease. Curr. Opin. Oncol. 24 (5), 495–505. PubMed PMID: 22729151; PubMed Central PMCID: PMCPMC6322210. 10.1097/CCO.0b013e328355e0f3 22729151 PMC6322210

[B56] Uppal T. Banerjee S. Sun Z. Verma S. C. Robertson E. S. (2014). KSHV LANA-the master regulator of KSHV latency. Viruses 6 (12), 4961–4998. PubMed PMID: 25514370; PubMed Central PMCID: PMCPMC4276939. 10.3390/v6124961 25514370 PMC4276939

[B57] Uppal T. Jha H. C. Verma S. C. Robertson E. S. (2015). Chromatinization of the KSHV genome during the KSHV life cycle. Cancers (Basel) 7 (1), 112–142. PubMed PMID: 25594667; PubMed Central PMCID: PMCPMC4381254. 10.3390/cancers7010112 25594667 PMC4381254

[B58] Vazquez Ede L. Carey V. J. Kaye K. M. (2013). Identification of Kaposi's sarcoma-associated herpesvirus LANA regions important for episome segregation, replication, and persistence. J. Virol. 87 (22), 12270–12283. PubMed PMID: 24006437; PubMed Central PMCID: PMCPMC3807934. 10.1128/JVI.01243-13 24006437 PMC3807934

[B59] Veesler D. Johnson J. E. (2012). Virus maturation. Annu. Rev. Biophys. 41, 473–496. PubMed PMID: 22404678; PubMed Central PMCID: PMCPMC3607295. 10.1146/annurev-biophys-042910-155407 22404678 PMC3607295

[B60] Wei F. Gan J. Wang C. Zhu C. Cai Q. (2016). Cell cycle regulatory functions of the KSHV oncoprotein LANA. Front. Microbiol. 7, 334. PubMed PMID: 27065950; PubMed Central PMCID: PMCPMC4811921. 10.3389/fmicb.2016.00334 27065950 PMC4811921

[B61] Yan L. Majerciak V. Zheng Z. M. Lan K. (2019). Towards better understanding of KSHV life cycle: From transcription and posttranscriptional regulations to pathogenesis. Virol. Sin. 34 (2), 135–161. PubMed PMID: 31025296; PubMed Central PMCID: PMCPMC6513836. 10.1007/s12250-019-00114-3 31025296 PMC6513836

